# Supplementary material for: Asymmetric Synthesis of Quaternary Hydantoins via a Palladium-Catalyzed Aza-Heck Cyclization
Source: J Am Chem Soc. 2025 Nov 14;147(49):44692–8. doi: 10.1021/jacs.5c16022 (PMC12703750; doi:10.1021/jacs.5c16022)
Supplement: Supplementary file 2 [file ja5c16022_si_002.zip › All NMR FID Files/S8 2D Spectra/S8 Conformer 1 HSQC/9/pdata/1/email_EM06-035-isomer#1-1HSQC_9_1.pdf]

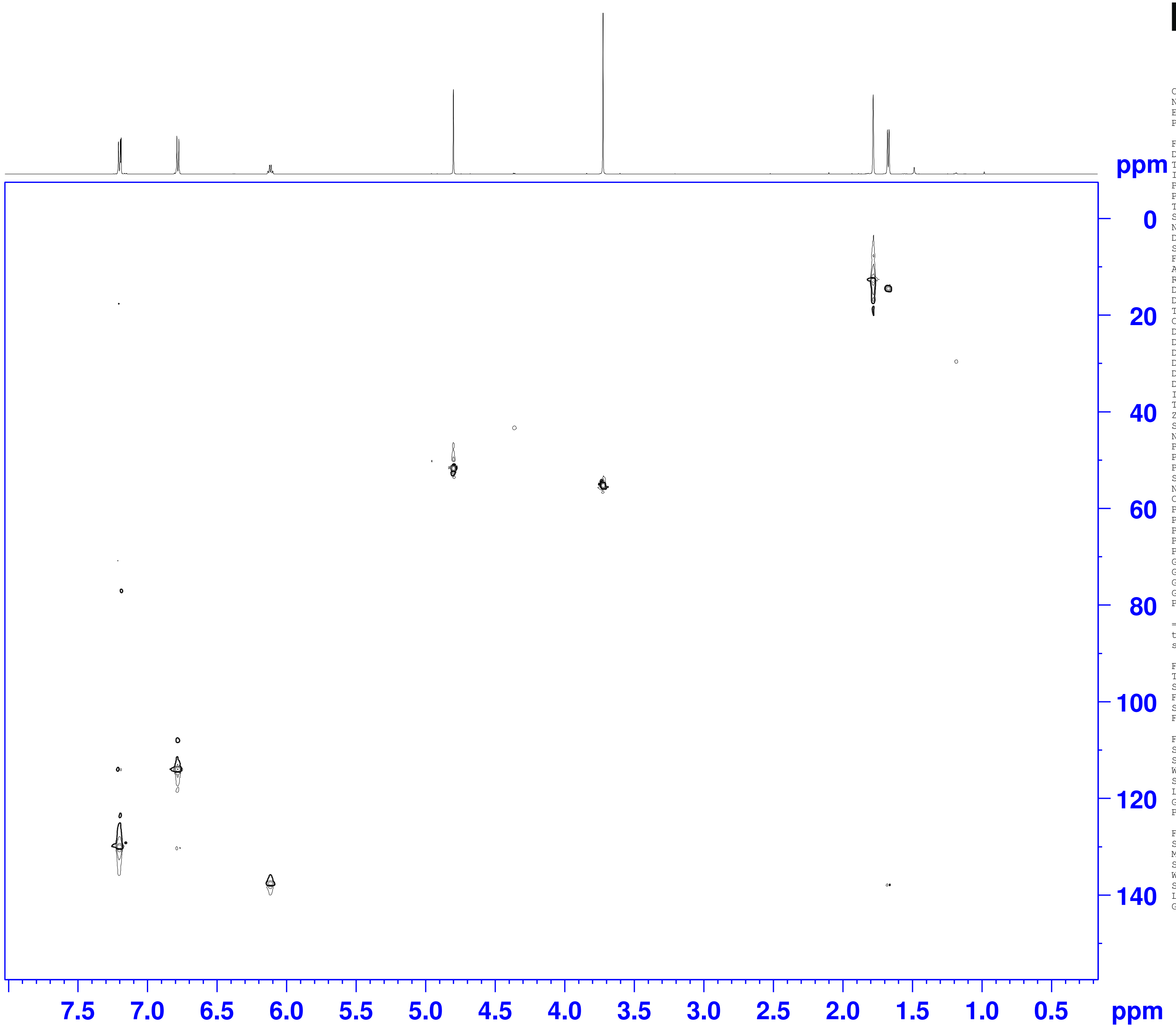


```
Current Data Parameters
NAME      EM06-035-isomer#1-1HSQC
EXPNO                      9
PROCNO                     1

F2 - Acquisition Parameters
Date_               20250211
Time                   14.30 h
INSTRUM   Avance Neo 600 at University of Delaware
PROBHD    Z185170_0001 (
PULPROG       hsqcedetgp
TD                      1024
SOLVENT                CDCl3
NS                         2
DS                        16
SWH                 4716.981 Hz
FIDRES              9.212853 Hz
AQ                 0.1085440 sec
RG                       101
DW                   106.000 usec
DE                     10.00 usec
TE                     298.0 K
CNST2            145.0000000
D0                0.00000300 sec
D1                1.50000000 sec
D4                0.00172414 sec
D11               0.03000000 sec
D16               0.00020000 sec
D21               0.00345000 sec
IN0               0.00002009 sec
TDav                       1
ZGOPTNS
SFO1             600.1325154 MHz
NUC1                      1H
P1                      8.07 usec
P2                     16.14 usec
PLW1              6.90000010 W
SFO2             150.9141262 MHz
NUC2                     13C
CPDPRG[2                garp
P3                     12.55 usec
P4                     25.10 usec
PCPD2                  60.00 usec
PLW2            106.00000000 W
PLW12             4.63759995 W
GPNAM[1]         SMSQ10.100
GPZ1                   80.00 %
GPNAM[2]         SMSQ10.100
GPZ2                   20.10 %
P16                  1000.00 usec

======== F1 INDIRECT DIMENSION ========
td1       256
sw_F1     165.000000

F1 - Acquisition parameters
TD                       256
SFO1                150.9141 MHz
FIDRES            194.537735 Hz
SW                   165.000 ppm
FnMODE        Echo-Antiecho

F2 - Processing parameters
SI                      1024
SF               600.1300575 MHz
WDW                    QSINE
SSB                        2
LB                         0 Hz
GB                         0
PC                      1.40

F1 - Processing parameters
SI                      1024
MC2            echo-antiecho
SF               150.9028085 MHz
WDW                    QSINE
SSB                        2
LB                         0 Hz
GB                         0
```
